# Experimental Study on Mechanical Properties of Different Resins Used in Oral Environments

**DOI:** 10.3390/medicina59061042

**Published:** 2023-05-28

**Authors:** Elena-Raluca Baciu, Carmen Nicoleta Savin, Monica Tatarciuc, Ioana Mârțu, Oana Maria Butnaru, Andra Elena Aungurencei, Andrei-Marius Mihalache, Diana Diaconu-Popa

**Affiliations:** 1 Department of Oral Implantology, Discipline of Dental Materials, Faculty of Dental Medicine, University of Medicine and Pharmacy “Grigore T. Popa”, 700115 Iași, Romania; 2 Department of Surgery, Discipline of Pediatric Dentistry, Faculty of Dental Medicine, University of Medicine and Pharmacy “Grigore T. Popa”, 700115 Iași, Romania; 3 Department of Oral Implantology, Removable Dentures and Technology, Faculty of Dental Medicine, University of Medicine and Pharmacy “Grigore T. Popa”, 700115 Iași, Romania; 4 Department of Surgery, Discipline of Basics of Physics and Biophysics in Dental Medicine, Faculty of Dental Medicine, University of Medicine and Pharmacy “Grigore T. Popa”, 700115 Iași, Romania; 5 Department of Machine Manufacturing Technology, “Gheorghe Asachi” Technical University of Iași, 700050 Iași, Romania; andrei.mihalache@tuiasi.ro

**Keywords:** removable dentures, acrylic resins, subtractive methods, additive technologies, finite element analysis

## Abstract

*Background and Objectives*: Acrylic resins remain the materials of choice for removable prosthesis due to their indisputable qualities. The continuous evolution in the field of dental materials offers practitioners today a multitude of therapeutic options. With the development of digital technologies, including both subtractive and additive methods, workflow has been considerably reduced and the precision of prosthetic devices has increased. The superiority of prostheses made by digital methods compared to conventional prostheses is much debated in the literature. Our study’s objective was to compare the mechanical and surface properties of three types of resins used in conventional, subtractive, and additive technologies and to determine the optimal material and the most appropriate technology to obtain removable dentures with the highest mechanical longevity over time. *Materials and Methods*: For the mechanical tests, 90 samples were fabricated using the conventional method (heat curing), CAD/CAM milling, and 3D printing technology. The samples were analyzed for hardness, roughness, and tensile tests, and the data were statistically compared using Stata 16.1 software (StataCorp, College Station, TX, USA). A finite element method was used to show the behavior of the experimental samples in terms of the crack shape and its direction of propagation. For this assessment the materials had to be designed inside simulation software that has similar mechanical properties to those used for obtaining specimens for tensile tests. *Results*: The results of this study suggested that CAD/CAM milled samples showed superior surface characteristics and mechanical properties, comparable with conventional heat-cured resin samples. The propagation direction predicted by the finite element analysis (FEA) software was similar to that observed in a real-life specimen subjected to a tensile test. *Conclusions*: Removable dentures made from heat-cured resins remain a clinically acceptable option due to their surface quality, mechanical properties, and affordability. Three-dimensional printing technology can be successfully used as a provisional or emergency therapeutic solution. CAD/CAM milled resins exhibit the best mechanical properties with great surface finishes compared to the other two processing methods.

## 1. Introduction

Complete and partial edentulism are the most severe and irreversible forms of edentation, which leads to imbalances in all elements of the stomatognathic system. This is why practitioners and researchers have been continuously concerned over time, seeking to find an optimal therapeutic solution that allows the restoration of all the affected functions.

Acrylic dental resins, especially poly-methyl-methacrylate (PMMA) resin, are commonly used in dentistry as denture base materials due to their advantages, including their relatively high strength, acceptable hardness, color stability, insolubility in the oral cavity, low weight, and low conductivity [1,2]. However, these materials have numerous drawbacks which are frequently reported by practitioners, the most common of which are poor mechanical strength, susceptibility to staining [3], curing shrinkage, and water sorption [3,4,5]. 

In the last decade, digital technologies have become an alternative to the conventional fabrication of acrylic removable dentures [6]. These methods substantially reduce workflow, facilitate quick communication between the dentist and the dental technician, and increase patient comfort [7]. Most available systems use subtractive and additive manufacturing to fabricate acrylic-based dentures [8,9]. The materials used in these technologies are industrially produced, so they have superior chemical and volumetric stability and optimal mechanical strength [10,11,12,13]. Subtractive systems use pre-polymerized acrylic discs, from which, based on the information received from the CAD unit, the future prosthesis will be milled. Milling strategies allow for obtaining a well-fitted prosthesis with highly accurate surfaces [14,15,16,17,18].

Several 3D printing technologies have been employed in dentistry, including stereolithography (SLA), digital light processing (DLP), selective laser sintering (SLS), selective laser melting (SLM), electron-beam processing (EBM), polyJet photopolymer printing, and fused deposition modeling (FDM). However, the stereolithographic printing of light-cured polymers has gained popularity [19].

Additive methods reduce workflow and the numbers of appointments, allow reproduction of all details, and reduce material waste [20,21].

Printing parameters, such as laser intensity, calibration of the printer and software, resin properties, build direction and angle, layer thickness and numbers, the bond between the layers, the amount of supporting structures, and post-polymerization conditions, play an important role regarding the quality of the final product [22]. It is a huge responsibility for dental staff to fabricate high-quality removable dentures, and it is important to know the details and particularities of each method. Digitalization in the prosthetics field has already activated research and clinical potential and will do significantly more in the near future. Consequently, these systems must be studied to determine their advantages and downsides. A removable denture requires a considerable number of clinical and technological procedures, as well as digital approaches that have the benefit of drastically lowering the amount of work required. On the other hand, the expenses of such a technical line are still rather high, and many experts do not view the procurement of equipment for producing detachable prostheses via additive or subtractive methods as a lucrative investment.

Finite element analysis (FEA) is a computational method that allows for the accurate modeling and analysis of complex structures, making it a valuable tool across a wide range of fields, from engineering to biology. Since Finite Element Analysis (FEA) is a numerical method capable of evaluating the stresses and deformations in structures. It has become a widely accepted and non-invasive approach for studying the biomechanics of biological systems and understanding the impact of mechanical forces on them [23].

Over the last few years, there has been rapid progress in the field of materials used for fabricating removable dentures. Nonetheless, the most recent studies on the mechanical and surface properties of resins do not cover the entire range of commercially available products across all countries. Our study is highly valuable for current practice as it analyzes the most used acrylic resins in our laboratories, replicating the real technological working flow involved in prostheses fabrication. The current research aimed to perform a comparative analysis of the selected mechanical characteristics of three types of resins used in conventional, subtractive, and additive technologies and to determine the optimal material and the most appropriate technology to obtain removable dentures with the highest mechanical longevity over time. Additionally, for ensuring the precision of the outcomes, we evaluated the ability of the FEA software to predict the direction of crack propagation, as well as provide valuable insights related to stress and strain phenomena.

The null hypothesis was that the mechanical and surface properties of the samples are unaffected by the fabrication process.

## 2. Materials and Methods

### 2.1. Study Design

**Figure 1:**
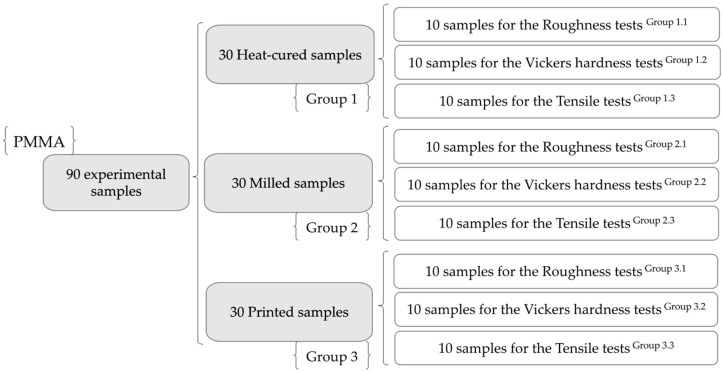
The experimental sample framework.

In order to investigate the properties of denture base materials, 90 samples (Figure 1) were manufactured using resins used for conventional and CAD/CAM subtractive- and additive-fabricated dentures. For the experimental tests that were conducted, the resin samples were divided up into three main groups, as shown in Table 1.

### 2.2. The Samples Design

**Figure 2:**
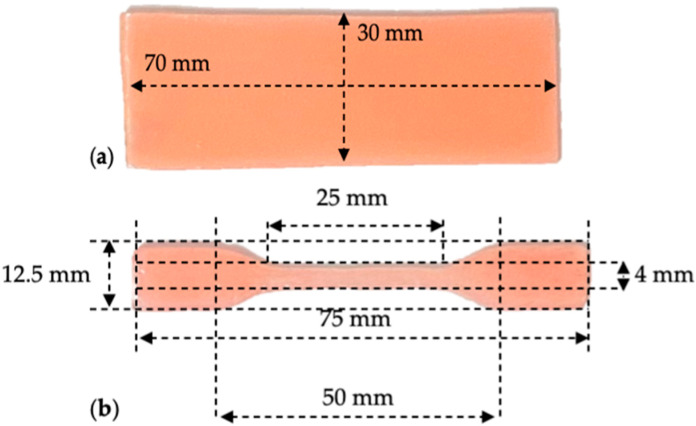
The design of the samples: (**a**) for the hardness and roughness tests; (**b**) for the tensile test.

For the hardness and roughness tests, rectangular samples with a width of 30 mm, a length of 70 mm, and a thickness of 2 mm were made (Figure 2a).

Dumbbell-shaped samples were used for tensile testing, with dimensions chosen according to ISO 527-2 [24] and ASTM D-638 [25] standards (Figure 2b).

### 2.3. PMMA Sample Realization

#### 2.3.1. Heat-Cured Samples

For the heat-cured samples, wax patterns were invested in Type 3 dental stones (Moldano, Kulzer GmbH, Hanau, Germany), respecting the manufacturer processing instructions: mixing a ratio of 100 g powder:30 mL water under a vaccum for 30 s.

Then, the metallic flask was immersed in 100 °C water for five minutes, the two sides of the flask were separated, the wax fragments were cleaned, and the mold was isolated using Isodent alginate solution (SpofaDental Inc., Jičín, Czech). The recommended mixing ratio for the resin preparation is 35 g powder to 14 mL liquid. The liquid was added to the mixing beaker along with an appropriate amount of powder, which was then stirred for one minute using a spatula. After 10 min, the Meliodent Heat Cure (Kulzer GmbH, Hanau, Germany) reached a packable consistency and could be manually pressed into the mold. The flask was immersed in boiling water for 15 min, after which the heat source was switched off. According to the manufacturer’s recommendations, a short cycle method was used in the polymerization process. The flask was allowed to cool slowly in a water bath after 20 min of boiling. Following polymerization, the specimens were extracted from the mold.

#### 2.3.2. Milled Samples

The wax patterns were scanned with a Swing DOF Scanner (DOF Inc., Seoul, Republic of Korea) and the computer-aided machine automatically milled (VHF K5 Plus, VHF, Ammerbuch, Germany) the experimental samples from CAD/CAM pink denture base resin discs (Polident, Polident d.o.o, Volja Draga, Slovenia), measuring 95 mm in diameter and 25 mm in thickness.

#### 2.3.3. Printed Samples

STL files were submitted to the digital light processing (DLP) printer (Asiga MAX, Asiga, Alexandria, NSW, Australia) for fabrication using Asiga DentaBASE resin (Asiga, Alexandria, NSW, Australia). After the printed samples were removed from the build platform, they were cleaned with 99% isopropyl alcohol and dried with steam.

Finally, a 20 min post-polymerization process was required, which was conducted using the Asiga Flash Post Curing Unit (Asiga, Alexandria, NSW, Australia).

#### 2.3.4. Finishing and Polishing

During the sample processing phase, we utilized the Acrylic Contouring & Finishing Kit HP (Shofu Dental GmbH, Ratingen, Germany) for denture finishing and polishing.

Initially, a dark gray AcryPoint coarse-grit BP1 tool (Shofu Dental GmbH, Ratingen, Germany) was utilized for 60 s (in dry conditions). Then, a brown AcryPoint medium-grit BP1 tool (Shofu Dental GmbH, Ratingen, Germany) was used for the same duration. The instruments’ rotational speed was rather modest at 10,000 rpm.

A light gray AcryPoints tool (Shofu Dental GmbH, Ratingen, Germany) and a gentle circular goat hair brush were employed at an even slower speed of 4000 rpm (60 s in dry conditions) for fine finishing.

For polishing, a Pala Polish Polishing paste (Kulzer GmbH, Hanau, Germany) was applied first to the samples, and then the procedure was carried out with a rag wheel (Kulzer GmbH, Hanau, Germany) three times for 60 s in order to achieve a flawless sheen.

### 2.4. Experimental Tests

#### 2.4.1. Roughness Tests

For each sample, six measurements (three records before and three records after finishing and polishing) were taken at the level of the examined surface using a contact-type roughness tester Form Talysurf^®^ (Taylor Hobson, Leicester, UK).

Based on the determined roughness parameter values, the influence of finishing on the micrometric profile of each surface (∆R_a_) was calculated as follows:

∆R_a_ = R_a before finishing_ − R_a after finishing_

R_a_—the arithmetic mean roughness.

#### 2.4.2. Vickers Hardness Tests

The Vickers tests were performed using a HVT-1000 automatic tester (Shanghai Daheng Optics and Fine Mechanics Co., Ltd., Shanghai, China) with a 50 gf load force for 10 s. On each sample, five determinations were made.

The Vickers hardness (HV) was calculated using the following formula:

HV = 1854.4 L/d^2^

L—the load force in gf; d—the average diagonal in µm.

#### 2.4.3. Tensile Tests

Standard tensile tests were conducted at room temperature in accordance with ISO 527-1: 2000 [25], and computer-controlled testing equipment was used for the direct strain measurements (Instron 2716-002, Instron, Norwood, MA, USA).

Young’s modulus and the tensile stress at yield were determined using a 1 mm/min crosshead speed.

Young’s modulus (E) was calculated according to Hook’s law:

E = σ/ε

σ—tensile stress (the amount of force applied per unit area, σ = F/A); ε—tensile strain (the extension per unit length, ε = dL/L).

#### 2.4.4. Finite Element Method (FEM)

**Figure 3:**
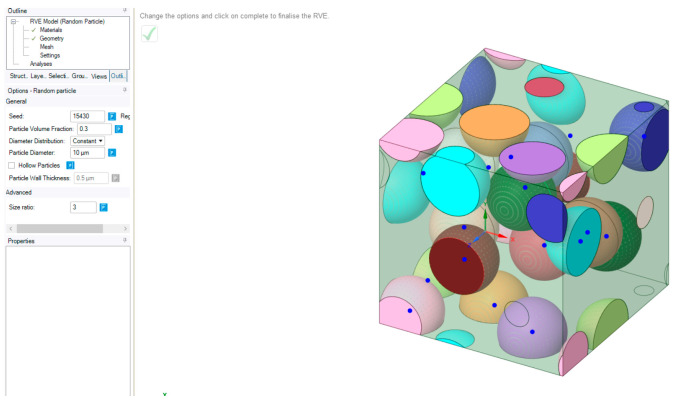
Material designer setup with graphical representations of the 3D view of the random particle type of arrangement with unprojected connections represented by blue dots.

The finite element method (FEM) goal was to model a fracture similar to the ones obtained after tensile tests that would give us stress and strain distributions, which are otherwise very difficult to measure. Our choice of software was Ansys (Ansys, Canonsburg, USA). First, we had to design the materials with mechanical properties similar to the ones used in our research. None of the three resins were available as a library source that could be used inside the simulation. Therefore, we used a specific tool -Ansys- as Material Designer. Poly-methyl-methacrylate (PMMA) resins are most commonly used for denture applications. We aimed to design materials similar to those used for mechanical testing [26]. For this purpose, we accessed the software library containing various types of materials, named Engineering Data. From the composite materials section we added a Polyamide resin and Epoxy E-Glass [27] for designing a materials similar to PMMA. In order to create this material, the software first requires a matrix and a binder and it was selected the resin as matrix and particles of epoxy as binder. The next step consisted of selecting a geometry for the inner arrangement at a macromolecular level. We chose a random particle distribution with default values of 15430 seed, a 0.3 particle volume fraction, and a 10 µm particle diameter. The diameter distribution was set to be constant. The resulting particle arrangement, and the mentioned settings, can be observed in Figure 3.

**Figure 4:**
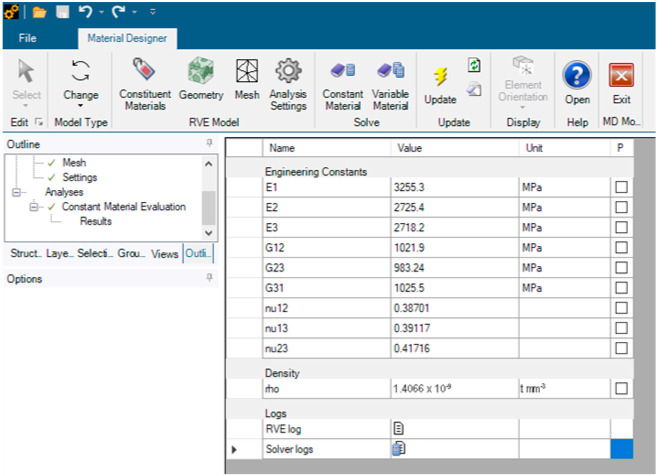
Material designer setup with graphical representations of the view of results that were obtained.

Mesh-wise, we chose a conformal and periodic meshing without imposing a maximum size for its elements. Our choice for anisotropy was orthotropic and we computed a linear elasticity property without the need for coefficients of thermal expansion or thermal conductivity. We used the periodic boundary conditions option for our analyses and chose a constant material type of solver, which we generically named PMMA. The software computed a Young’s modulus of 3255.3 MPa on XY, 2725.4 MPa on YZ, and 2718.2 MPa on XZ. We obtained a Poisson’s ratio of 0.38701 in XY, 0.39117 in YZ, and 0.41716 in XZ. The density was reported as 1.4066 × 10^−9^ t mm^−3^ (Figure 4).

The newly designed material needed for finite element analyses was imported into the Engineering Data section of a Static Structural type of analysis. In order to obtain a fracture direction, the sample was designed with the same geometric dimensions as the ones specified by standards in a 3D software solution (Siemens SolidEdge academic, Siemens, Munich, Germany) and used inside experimental tensile tests. Based on the results of the tensile tests, we designed a pre-crack about the same height as the area where the fracture appeared after tensile testing. The 3D model was later translated into a Parasolid file that was easily interpreted by the software.

By reading the internal library, namely Engineering Data, we were able to assign the newly created material to the imported geometry. Because of the complexity of this process, we used a virtual topology tool based on an edge mapping type of behavior. Our choice for predicting a crack direction was to use the SMART crack growth tool available in Ansys. This was suitable for our setup because crack growth simulation expands in homogenous environments, which was the case for our specimen. The criterion is in fact a rate of energy release type of method, which uses stress intensity factors (SIFs) for assessments. We chose a static crack type of growth, which calculates the extension of each front node of the crack. We had to generate a new coordinate system specific to the crack itself, where the X points to the direction of propagation and the Y sits almost normal to its upper face. Mesh-wise, we imposed a patch conforming method that uses quadratic element orders and a face meshing method for both of the crack’s faces with an element size of just 0.25 mm, as the mesh general settings were set to 0.5 mm with an aggressive mechanical type of error limits assessment. That resulted in 9590 elements and 18,210 nodes (Figure 3). The fracture tool included a pre-meshed crack with six solution contours and the SMART crack growth tool was set to be static. The chosen option for failure criteria was a stress intensity factor with a critical rate of 100 MPa·mm^(0.5)^. The analysis settings received a 0.001 sec end time defined by ten sub-steps. Conditions were set in the form of a fixed support on the lower face of the sample and a force solicitation on the upper face (Figure 4).

**Figure 5:**
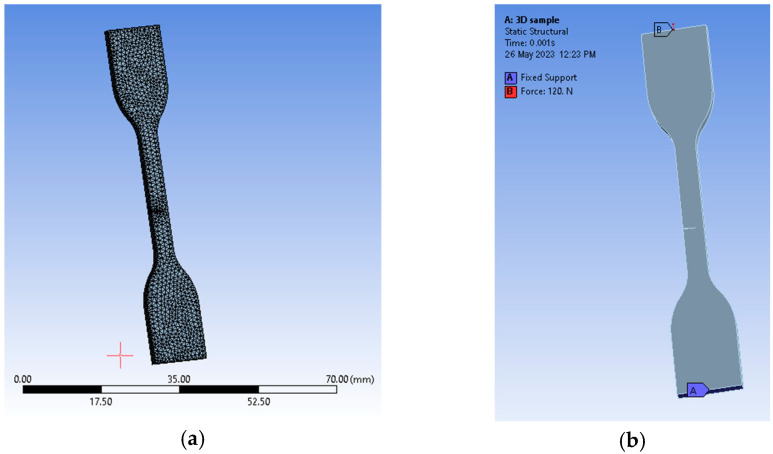
Setup with graphical representations of the (**a**) 3D view of the meshed body and (**b**) 3D view with supports and conditions applied on the sample body.

Equivalent elastic strain registers showed values a little over 0.008 mm/mm (Figure 5a), as the propagation direction was similar to the one observed in our tested samples and the equivalent stress, evaluated by means of von Mises criteria, reached 1634.8 MPa along the fracture line (see Figure 5b). The analysis was stopped just before the body was separated.

### 2.5. The Statistical Analysis

For the statistical data analysis, Stata 16.1 software (StataCorp, College Station, TX, USA) was used. The one-way ANOVA analysis was employed to check if there were statistical differences in terms of surface roughness, hardness, and tensile parameters across the three types of resins used. Furthermore, the Bonferroni multiple comparison test was used to check where the statistically significant difference lay by zooming in on each of the possible paired samples. The statistical analysis was carried out with a significance level of *p* < 0.05.

## 3. Results

### 3.1. Evaluation of the Roughness Parameters

The findings from the statistical analysis for the compared roughness parameters are shown in Table 2.

**Figure 6:**
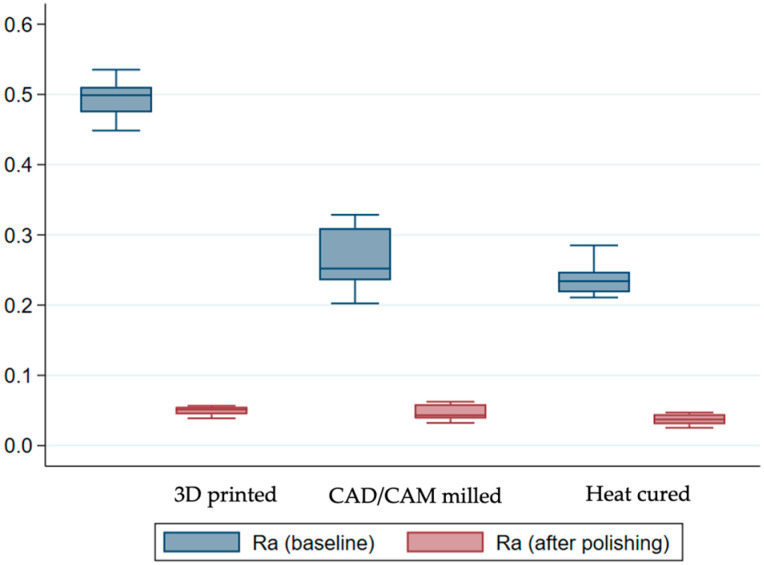
Box plot of surface roughness by resin before/after polishing.

The results of the roughness coefficients before finishing and polishing revealed a statistically significant difference between the three groups (*p* < 0.001). However, the Bonferroni multiple comparison test indicated that only the 3D printed samples were, on average, different compared to the other two groups (*p* < 0.001). As is also displayed in Figure 6, on average, the heat-cured sample displayed the lowest roughness coefficients.

As expected, after polishing and finishing, the average roughness coefficients dropped in all three methods, with the heat-cured sample still reporting, on average, the lowest coefficients. The ANOVA confirmed that there was still a statistically significant difference in roughness between the groups (*p* = 0.0112). Nonetheless, the difference is only significant between the 3D printed and the heat-cured samples, as indicated by the Bonferroni test (*p* = 0.011). Finally, when investigating the differences in terms of averages across the study groups, the results confirmed a significant difference between them (*p* < 0.001).

### 3.2. Evaluation of the Vickers Hardness

At a magnification of 400:1, Figure 7a–c illustrates the indentations produced by the indenter on the surface of the samples subjected to Vickers hardness analyses.

The average values of the determinations are presented in Table 3, with the 3D printed sample reporting, on average, the smallest HV coefficients.

**Figure 8:**
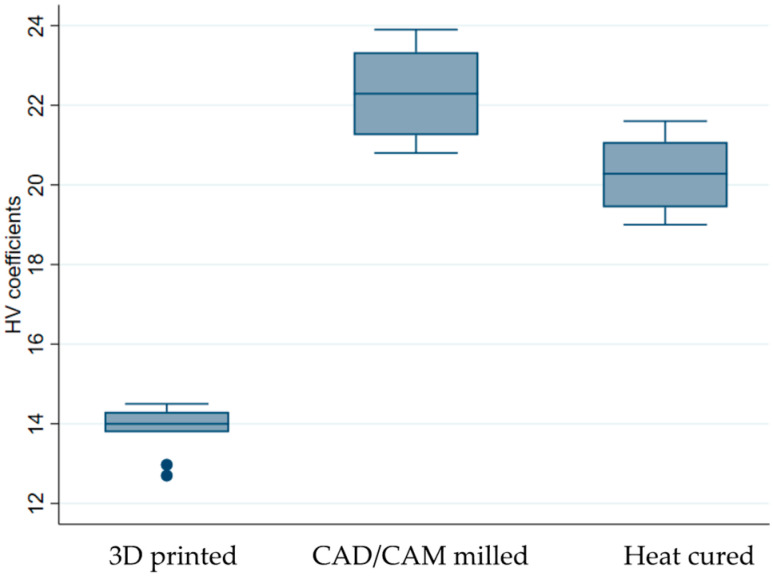
Box plot of Vickers hardness values by resin used.

When looking at the distribution of our samples, the CAD/CAM milled sample clearly displays, on average, the highest Vickers. At the other end lies the 3D printed sample, with the lowest values (Figure 8).

The one-way ANOVA test revealed that the surface hardness depends on the type of resin used determine [F(2.27) = 251.63, *p* < 0.001]. According to the Bonferroni multiple comparison test, there was a statistically significant difference in the mean values of the HV coefficients for all potentially matched samples (*p* < 0.001).

### 3.3. Evaluation of the Tensile Parameters

The average values of the modulus and tensile stress at yield obtained by the tensile test are shown in Table 4.

**Figure 9:**
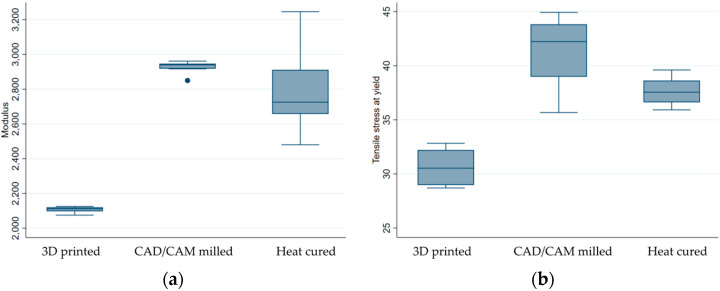
Box plot of mechanical characteristics obtained by tensile tests by resin used: (**a**) modulus and (**b**) tensile stress at yield.

According to the descriptive results, the CAD/CAM samples group displayed greater modulus coefficients, as well as higher tensile stress parameters (Table 4 and Figure 9).

The one-way ANOVA also revealed a significant difference (*p* < 0.001) in the mechanical characteristics between the three groups (*p* < 0.001). Zooming in to each of the possible pairs, the Bonferroni test revealed that, when it comes to the modulus coefficient, only the 3D printed samples were, on average, different from the other two samples (*p* < 0.0001). However, when looking at the tensile strength parameter, a statistically significant difference between the mean values was confirmed for all three possible paired samples.

### 3.4. Finite Element Method (FEM) Analysis

**Figure 10:**
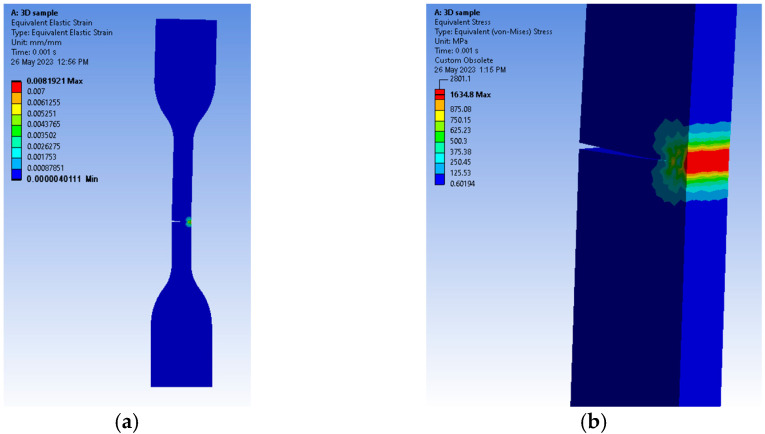
Graphical representations of FEM results: (**a**) front view of the equivalent elastic strain distribution along the fracture line and (**b**) semi-side view of the equivalent (von Mises) stress distribution along the fracture line.

The contours of the samples showed that the critical points were at the ends of the fracture line and all along its length as the crack propagated. This type of result (Figure 10 a,b) is similar to the one observed in a real-life specimen that was subjected to a tensile test.

## 4. Discussion

Based on the findings of this research, there were significant differences between the sample groups for all the characteristics investigated, indicating that the study’s null hypothesis was rejected. Although 3D-printed samples recorded the highest surface roughness, heat-cured samples and CAD/CAM milled samples showed the lowest Ra coefficients, both before and after polishing. This discrepancy could be related to the manufacturing process of pre-polymerized resin blocks in subtractive technologies, as well as the reduced degree of polymerization in 3D printing.

Similar results were obtained by Helal et al. [28], who found significant differences between the tested denture base resins (3D-printed, CAD/CAM milled resin, and polyamide flexible resins). These findings are also supported by those obtained by the authors Zeidan et al. [29]. In contrast, Srinivasan et al. [18] demonstrated that the surface roughness of milled and rapidly prototyped resins was similar.

Srinivasan et al. [4] conducted a study in which the surface roughness of CAD/CAM milled and rapidly prototyped/3D-printed resins utilized in the fabrication of complete dentures was evaluated using a high-resolution white light non-contact laser profilometer (CyberSCAN CT 100, Cyber technologies, Eching-Dietersheim, Germany). Based on their findings, the authors concluded that both types of resins exhibited similar levels of surface roughness.

Contact testers, such as stylus profilometers, directly measure the height of surface irregularities and can provide highly accurate results. However, they can cause damage to delicate surfaces, are sensitive to vibrations, and require a high level of operator skill to use properly. On the other hand, non-contact testers, such as laser and optical profilometers, are non-invasive, can measure surface roughness at high speeds, are able to measure smaller asperity than contact types, allow for simultaneous observations of surface images and height profiles (microscope type), and are able to acquire high-definition, fully focused images that rival those of SEMs (color 3D laser microscopes) [30]. However, they can be more expensive, require complex alignment procedures, the measurement area is limited, and they are used for in-situ measurements [31].

The most significant effect of finishing on the micrometric profile was observed on 3D-printed (∆Ra = 0.445 ± 0.027 µm) sample surfaces, whereas the impact was comparable in the CAD/CAM milled and heat-cured samples. Even though polishing improved the samples, they remained in the same order, with the 3D sample having the highest coefficients.

In research conducted by Freitas et al. [32], the surface properties of milled dentures were found to be comparable to those of heat-polymerized denture base resins, but with reduced *Candida albicans* adhesion. According to other authors, even after proper finishing and polishing and long-term thermocyclic aging, traditional complete dentures were associated with greater *Candida* adhesion than CAD/CAM milled prostheses.

Al Moaleem et al. [33] and Ramage et al. [34] reached the conclusion that superior denture surface characteristics, such as porosity and surface roughness, contribute to a reduction in microbial *Candida* adherence, lowering the incidence of denture stomatitis.

In their research, Paolone et al. [35] emphasize the importance of denture surface quality in reducing bacterial plaque adherence and determining superior esthetics and excellent clinical results.

Regarding the mechanical parameter of hardness and tensile parameters, there were statistically significant differences between the groups.

Vickers hardness measurements revealed elevated values for the CAD/CAM milled experimental samples and the lowest values for the 3D-printed samples. The conventional heat-cured resin group recorded an average hardness value. Comparable findings were revealed in recent studies [6,36].

The one-way ANOVA revealed a significant *p*-value when examining the statistical differences between the mean modulus and tensile stress values of the three groups (*p* 0.001). Comparing each of the potential pairs, the Bonferroni test revealed that, in terms of the modulus coefficient, only the 3D-printed samples differed from the other two on average (*p* 0.0001). However, when examining tensile stress, a statistically significant difference between the mean values was confirmed for all three potential matched samples.

The internal structures of materials determine their mechanical qualities, such as hardness and flexural strength [33]. CAD/CAM milled polymers have a higher flexural strength than denture bases that are manufactured with compression molding, according to studies [30].

According to Srinivasan et al. [8], milled resins have a higher ultimate strength, toughness, and elastic modulus than printed resins, whereas Al-Dwairi et al. [11] found that CAD/CAM milled denture base resins have a considerable advantage in surface wettability, surface roughness, and surface hardness.

Many studies have indicated that milled removable dentures demonstrate greater flexural strength, flexural modulus, and impact strength than typical heat-cured groups [37,38,39,40,41,42], as well as superior surface qualities compared to 3D-printed and conventional ones.

Acrylic resins for rapidly prototyped removable dentures have a poor double-bond conversion compared to standard heat-curing resins, which can impact their mechanical qualities. Unlike CAD/CAM milled resins, high pressure promotes the creation of longer polymer chains and can decide a greater degree of monomer conversion [37,38,39,40,41,42,43,44]. The lower characteristics of 3D-printed resin may also be attributed to the processing technique used, such as light polymerization and printing methods (e.g., the layering technique and printing orientation) and parameters.

Steinmassl et al. [45] obtained contradictory results, indicating that digital dentures do not generally have a higher fracture resistance compared to conventional prostheses made of self-curing or heat-curing resins. All CAD/CAM milled denture base resins have greater elastic moduli than heat- or self-curing resins. The fracture surface analyses imply that the microstructure of the resin, as opposed to the polymer chain length, which would be affected by the curing technique, may be accountable for the differing mechanical properties. They believe the changes in mechanical properties are due to the composition of the resin rather than the industrial procedure used [46].

According to the literature, CAD/CAM milled dentures have a superior fit and greater surface qualities and mechanical properties compared to traditional heat-polymerized dentures. Nonetheless, removable dentures that are fabricated with heat-cured resins remain a viable alternative since the mechanical characteristics and surface quality of these materials allow the manufacture of clinically well-tolerated prostheses. Being in direct contact with the oral tissues, all the new materials must be accurately tuned to fit the requirements and usage conditions [47].

The FEA software predicted the correct propagation direction and offered valuable information in a graphical manner, which can be used for future research. However, the authors acknowledge the need for further refinement since our material mechanical properties may be different depending on the producer’s manufacturing process.

Due to the lack of proper understanding regarding the biomechanical principles of the materials involved in restorative procedures, many detrimental effects resulted, causing restorative failures. Therefore, in order to know the behavior of the materials and dental tissues, biomechanical studies are very crucial [6,7].

The limitations of the current research were the lack of denture base materials, the absence of oral condition simulations, and that the samples were not aged by thermocycling. Given these constraints, further in vitro and in vivo studies are required.

## 5. Conclusions

The study was based on three different groups of specimens which gave us 90 samples to test. Every ten samples had two more that were used for validation purposes. Each sample proved consistent within limits, thus proving our results. Out of the three samples, the 3D-printed samples experienced the lowest surface quality in terms of surface finishing and structural integrity. CAD-CAM milled and heat-cured resins have comparable roughness parameters; however, the milled samples exhibited better mechanical properties than conventional and 3D-printed resins.

Finite element analyses predicted similar behaviors in terms of crack propagation, as real-life samples showed similar results if the concentrator was placed in the same area. Statistically, ANOVA highlighted different values for the 3D-printed samples, on average, compared to the others. Despite the advantages of CAD/CAM milling technology, heat-cured resins continue to be a feasible option since their surface quality and mechanical properties allow the fabrication of satisfactorily removable dentures with long-term durability.

Although 3D-printed removable dentures can be successfully used as a provisional or emergency therapeutic solutions, further refinement is still required in order to make them suitable for prosthetic constructions with great longevity over time. The authors acknowledge the need for further refinement of the results and a wider research base.

## Figures and Tables

**Figure 7:**
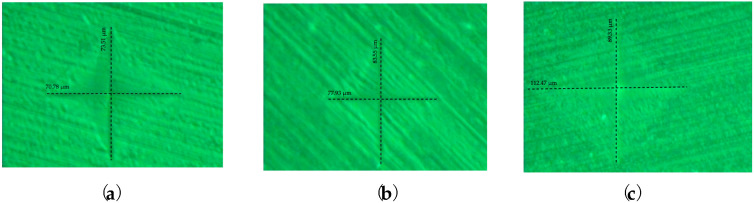
The impressions of the penetrator on the surface of the samples: (**a**) Group 1.2—Sample 3 heat-cured; (**b**) Group 2.2—Sample 2 CAD/CAM milled; (**c**) Group 3.2—Sample 8 3D printed.

**Table 1:** Resin groups with their product information and experimental equipment.

Resin Groups	Resin	Experimental Equipment		
		Surface Roughness	Vickers Hardness	Tensile Tests
Group 1 Heat-cured group	Meliodent Heat Cure (Kulzer GmbH, Hanau, Germany)	Form Talysurf^®^ tester (Taylor Hobson, Leicester, UK)	HVT-1000 (Shanghai Daheng Optics and Fine Mechanics Co., Ltd., Shanghai, China)	Instron 2716-002, Instron, Norwood, MA, US
Group 2 Subtractively manufactured group	Polident (Polident d.o.o, Volja Draga, Slovenia)			
Group 3 Additively manufactured group	Asiga DentaBASE resin (Asiga, Alexandria, NSW, Australia)			

**Table 2:** The roughness parameter values for the studied samples.

Group	Number of Samples	Mean Values ± Standard Deviation		
		Ra before Finishing and Polishing [µm]	Ra after Finishing and Polishing [µm]	∆Ra [µm]
Heat cured	10	0.239 ± 0.024	0.037 ± 0.008	0.201 ± 0.022
CAD/CAM milled	10	0.267 ± 0.044	0.046 ± 0.011	0.221 ± 0.035
3D printed	10	0.494 ± 0.028	0.050 ± 0.007	0.445 ± 0.027
F(df)		177.24 (2,27)	5.33 (2.27)	224.11 (2.27)
*p*-value		0.000	0.0112	0.000

A one-way ANOVA was used. The significance level was established at 5%.

**Table 3:** Vickers hardness values for the studied samples.

Group	Number of Samples	Mean Values ± Standard Deviation
		Vickers Hardness HV
Heat cured	10	20.257 ± 0.854
CAD/CAM milled	10	22.301 ± 1.115
3D printed	10	13.853 ± 0.586
F(df)		251.63(2.27)
*p*-value		0.000

A one-way ANOVA was used. The significance level was established at 5%.

**Table 4:** The average values of the mechanical characteristics of the experimental samples obtained by tensile tests.

Group	Number of Samples	Mean Values ± Standard Deviation	
		Modulus (Segment 0.0005–0.0025 mm/mm) [MPa]	Tensile Stress at Yield (Offset 0.5%) [MPa]
Heat cured	10	2805.779 ± 245.604	37.575 ± 1.272
CAD/CAM milled	10	2930.298 ± 32.013	41.188 ± 3.449
3D printed	10	2106.551 ± 16.663	30.642 ± 1.501
F(df)		95.98(2.27)	54.66(2.27)
*p*-value		0.000	0.000

A one-way ANOVA was used. The significance level was established at 5%.

## Data Availability

Not applicable.
